# Case of Purulent Infection

**Published:** 1850-05

**Authors:** J. H. Weir

**Affiliations:** Philadelphia


*Case of Purulent Infection. Service of* M. Roux. *Hotel Dieu.* Reported by J. H. Weir, M. D., Philadelphia.

Julien, aged 21 years, had received a blow on the frontal region by falling against the corner of a door. He entered the hospital St. Antoine, where he remained for eight days. No symptoms of compression of the brain appeared after the accident, and the small wound cicatrized rapidly. Six days after leaving the hospital, an abscess formed at the lower part of the cicatrix; this was opened, and the patient then entered the Hotel Dieu on the 30th of April.

*April* 30. Abscess situated on a level with the left frontal protuberance, an inch in length, and about one half in breadth. Suppuration commenced freely; the bone was soon exposed; the fever became intense, with little appetite. Ordered simple dressing, with absolute diet.

During the following six days the fever greatly abated; the pus was healthy, the integuments surrounding the wound were not inflamed, and the appetite was better.

On the night of the seventh, the dressings of the wound became detached, so that the wound was exposed to the air for the space of six or seven hours. Soon after, the patient was seized with a chill, dry heat of skin, vomiting of bilious matter, pain between the shoulders, *and the abscess ceased discharging pus.*

*May 8th.* The vomiting still continues; the patient in a low state; abscess nearly dry; pulse small and frequent; skin hot and dry; the pain between the shoulders has greatly augmented. On percussion, dull sound at the base of right lung, respiratory murmur absent at this point. Pleurisy diagnosed with symptoms of purulent infection. Tartar emetic grs. iii.

*May 9th.* Patient complains of a severe pain in the region of the liver, accompanied with great weakness.

*May 10th.* Blister to right hypochondrium. Patient about the same. Surface of abscess glazed and dry.

*May* 11*th*. No new phenomena have occurred; there is greater prostration; pulse very small; severe pains in the region of the liver, accompanied with a dry, painful cough; diarrhœa; icteric tinge of the skin.

*May* 12*th*. Patient continued to sink up to 12 o'clock, when he expired.

*Autopsy.*—An icteric tinge of the skin over the whole body; the wound of the forehead is enlarged, and the periosteum is detached; no fracture of the cranium; surface of the coronal suture rugose, bathed in pus. On examining the internal surface of the bone, it was found to be denuded, its greyish color and its rugosities show that there has been effusion of pus from the exterior to the interior. The dura-mater beneath the wound is of a black color, slightly softened and soaked in pus; no false membranes above the arachnoid; slight injection of the surface of the brain; the cerebral substance is of a normal consistence and color.

Pleurisy of the right side with exudation of a troubled liquid; pseudo-membrane enveloping the lower lobe of the right lung; underneath this membrane were found several superficial metastatic abscesses; no induration of lung; some metastatic abscesses in the left lung. The brain was healthy throughout. In the interior of the spleen three metastatic abscesses were found; one of them in process of suppuration. Stomach and intestines generally healthy. Jugular veins and the sinuses of the dura-mater contained some black clots, slightly consistent; these were found without any traces of pus.

The above is one among the many cases (collected during a residence in Paris of some two years) of death occurring from nothing but that insidious and most fatal sequence of suppuration, purulent infection, or metastatic abscesses. It has been my desire to give a report of such cases only in which the powers of this "purulent infection" are most evident. In reading the case just reported, any one will be struck with the fact, that up to the night of the 7th May, the patient had continued to do well; that during that night the abscess was left exposed to the action of the air for several hours, and that from that time the unfavorable symptoms, which ended in the speedy death of the patient, date themselves. Previous to the exposure, the abscess suppurated freely; immediately afterwards the discharge ceased, and so continued till death.

The general health of the hospital Hotel Dieu, at the time of the patient's death was good. Most of the operations performed at this time progressed favorably, unmarked by any peculiar symptoms, so that we cannot charge the hospital as the exciting cause of the man's death. We are, therefore, brought to the conclusion that it was something distinct and individual that caused this fatal termination to a very slight injury ; and we are led to believe, from the history of the case, that the exposure of the suppurating surface of the abscess to the air, thereby altering the nature of the pus, was the exciting and effectual cause of the death of the patient.

